# Empirical Modelling of Hydrodynamic Effects on Starch Nanoparticles Precipitation in a Spinning Disc Reactor

**DOI:** 10.3390/nano10112202

**Published:** 2020-11-04

**Authors:** Sahr Sana, Vladimir Zivkovic, Kamelia Boodhoo

**Affiliations:** School of Engineering, Merz Court, Newcastle University, Newcastle Upon Tyne NE1 7RU, UK; s.sana@newcastle.ac.uk (S.S.); vladimir.zivkovic@newcastle.ac.uk (V.Z.)

**Keywords:** spinning disc reactor, nanoparticles, solvent–antisolvent precipitation, empirical model, Reynolds number, rotational Reynolds number, Rossby number

## Abstract

Empirical correlations have been developed to relate experimentally determined starch nanoparticle size obtained in a solvent–antisolvent precipitation process with key hydrodynamic parameters of a spinning disc reactor (SDR). Three different combinations of dimensionless groups including a conventional Reynolds number (*Re*), rotational Reynolds number (*Re_ω_*) and Rossby number (*Ro*) have been applied in individual models for two disc surfaces (smooth and grooved) to represent operating variables affecting film flow such as liquid flowrate and disc rotational speed, whilst initial supersaturation (*S*) has been included to represent varying antisolvent concentrations. Model 1 featuring a combination of *Re*, *Re_ω_* and *S* shows good agreement with the experimental data for both the grooved and smooth discs. For the grooved disc, *Re* has a greater impact on particle size, whereas *Re_ω_* is more influential on the smooth disc surface, the difference likely being due to the passive mixing induced by the grooves irrespective of the magnitude of the disc speed. Supersaturation has little impact on particle size within the limited initial supersaturation range studied. Model 2 which characterises both flow rate and disc rotational speed through *Ro* alone and combined with *Re* was less accurate in predicting particle size due to several inherent limitations.

## 1. Introduction

Nanoparticles are incredibly small particles with sizes ranging between 1 and 100 nm. They are known for their unique chemical and physical properties which make them stand out when compared to their larger counterparts. These properties may be intrinsic, such as magnetic, optical or electronic; or extrinsic, such as shape or a high surface area [1]. Consequently, nanoparticles have potential applications in various fields. One such example is of starch nanoparticles which have several medical and industrial applications as, for instance, polymer drug carriers [2,3,4,5], reinforcements in nanocomposites [6], absorbent in wastewater treatment [7], binder in paper manufacturing [8] and as a packaging component [9,10,11]. Starch nanoparticles can be created through either top-down (e.g., hydrolysis or physical methods), or bottom-up processes (precipitation). A few of these techniques are highlighted in Table 1 alongside details of starch nanoparticles sizes obtained through each respective technique. Each of these techniques results in nanoparticles with distinguished characteristics to suit its application. For example, starch nanoparticles produced through acid hydrolysis tend to be crystalline in nature and have greater thermal and colloidal stability [12]. On the other hand, amorphous starch nanoparticles are often used in drug delivery systems as they take the form of a V-type polymorph, impeding digestion, whereas the A-type structure found in nanocrystals is readily digestible [13]. A widely applied method of generating starch nanoparticles is solvent–antisolvent precipitation. It is often cheaper, environmentally safer and more efficient than top-down process such as milling or hydrolysis [14,15]. Solvent–antisolvent precipitation is a bottom-up technique, involving the dissolution of the solute (starch) in a solvent (e.g., a solution of sodium hydroxide), followed by the addition of an antisolvent (e.g., ethanol) for the creation of supersaturation to precipitate out solid particles [16]. An alternative to the sodium hydroxide solvent is dimethylsulphoxide (DMSO), which is preferable when dealing with pH sensitive systems [17]. Alongside using DMSO as the solvent, Wu et al. (2016) studied the antisolvent ability of various alcohols, revealing a decline in particle size with shorter chained alcohols [17]. It was further reported by the authors that whilst the ratio of antisolvent to solvent played a key role in determining the morphology of the starch nanoparticles, it had very little effect on the particle size. Chin et al. (2011), also carried out a similar investigation, varying the ratio of the ethanol antisolvent to the NaOH solvent [18]. The results indicated a change in morphology of the starch nanoparticles from fibrous structures to spherical nanoparticles at increased antisolvent concentrations, as well as a reduction in particle size.

Other than the ratio or type of solvent/antisolvent, mixing intensity is another fundamental parameter in solvent–antisolvent processes to aid in the integration of the anti-solvent into the solute/solvent mixture and maintain constant supersaturation [29]. For the production of small particles with a narrow size distribution, it is vital that process mixing, especially mixing at the molecular scale or micromixing, is faster than the precipitation process. Many devices which have been studied for solvent–antisolvent precipitation processes can provide intensified mixing with low mixing times. These include confined impingement jet reactors (CIJR) [30], microreactors [31,32,33,34], oscillatory baffled reactors (OBR) [35,36] and rotating packed beds (RPB) [37,38]. For instance, Valente et al. (2012) investigated the scale-up of confined impinging jet mixers (CJIM) for the solvent–antisolvent precipitation of polymer nanoparticles [39]. The authors related particle size to various operating conditions including solvent–antisolvent ratio, polymer concentration, jet velocity and mixing conditions. Dimensionless parameters Reynolds number and Damköhler number were applied to correlate particle size. However, the authors concluded that Damköhler number, which is the ratio of mixing time to particle formation time, could be used to account for the interactions between process hydrodynamics and precipitation but was not very suitable in the scale-up of CJIMs. Reynolds number on the other hand was considered an important parameter in the determination of particle size.

The current work concerns the precipitation of amorphous starch nanoparticles through the solvent–antisolvent precipitation method in a spinning disc reactor (SDR). The SDR is an effective process intensification technology which has successfully been applied for the production of nanoparticles through methods involving reactive precipitation [40,41,42,43,44,45,46,47,48,49,50] and solvent–antisolvent precipitation [51,52]. By means of rotation, the large centrifugal forces produced by the disc encourage the formation of thin liquid films with thicknesses usually around 50 to 300 microns for water-like liquids [44,53]. Within these thin liquid films, waves and instabilities are created as a result of the high shear generated through the rotation of the disc, intensifying micromixing within the film [54,55,56]. Plug flow characteristics have also been attributed to film flow in the SDR [57]. Furthermore, residence times are short (of the order of seconds in one disc pass) and can be controlled through the manipulation of operating parameters.

The characteristic parameters for the SDR, such as average radial velocity, *u*_av_, (Equation (1)) film thickness, δ (Equation (2)) and residence time, *t*_res_ (Equation (3)) are derived from a model based on the Nusselt (1916) theory [55,58].

(1) uav = Q2ω212π2νr13

(2) δ = 3Qν2πω2r213

(3) $$t_{\mathrm{res}}\;=\;\frac{3}{4}{\left(\frac{12\pi^{2}\nu }{Q^{2}\omega^{2}}\right)}^{1/3}\left(r_{0}^{4/3}-r_{i}^{4/3}\right)$$

where *Q* is the volumetric flow rate, *ω* the angular velocity, ν is the kinematic viscosity of the liquid, *r_o_* is the outer disc radius and *r_i_* is the inner disc radius where the feed is introduced to the SDR.

The validation of these equations relies largely on the dominance of centrifugal forces. When centrifugal acceleration dominates, Coriolis acceleration is considered to be negligible. This is the case typically for highly viscous liquids and/or at distances away from the centre of the disc where film thickness is at a minimum, satisfying the following condition for the centrifugal model [59]:

$$\nu \gg \omega \delta^{2}.$$

**Figure 1:**
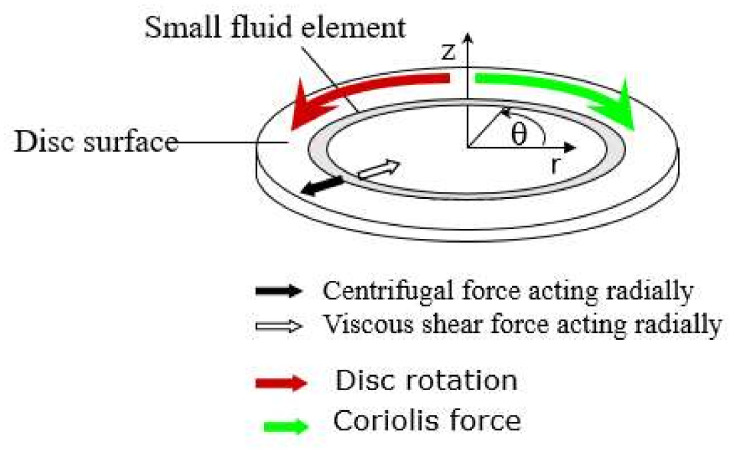
Schematic diagram showing the centrifugal and Coriolis forces acting on a rotating disc.

Coriolis forces (Figure 1), on the other hand, come into play when the radial velocity distribution term, *v*_r_, is of a considerable magnitude. This generates acceleration in the angular direction opposite to rotation, known as Coriolis acceleration, and is defined as [55]:

(4) $$a_{\mathrm{cor}}=2v_{r}\omega .$$

Previous work involving the solvent–antisolvent precipitation of starch nanoparticles in a spinning disc reactor has demonstrated that starch nanoparticle size is influenced by flow rate, disc rotational speed, and antisolvent to solvent ratio [28]. The study indicated a reduction in particle size with an increase in flow rate and disc rotational speed. This was attributed to the increase in shear as either flow rate or disc rotational speed were increased, leading to enhanced micromixing between the solvent/solute and the antisolvent, and generating supersaturation at a faster rate. In addition, an increase in antisolvent to solvent ratio demonstrated a reduction in particle size, caused by an increase in supersaturation.

The interactions of these three single variables have been further analysed in the present work through empirical correlations consisting of the relevant dimensionless numbers derived to predict starch nanoparticle size for smooth and grooved discs. Various dimensionless numbers relevant to the SDR and the solvent/antisolvent process are first introduced, followed by the development of the empirical correlation using experimental results from the aforementioned work by the authors. Through the use of dimensionless numbers, the intention is to reduce the number of independent variables as well as the data used to acquire the empirical model. Besides relating the operating parameters to starch nanoparticle size obtained in the SDR, the aim of such a model is to highlight the significance of each of the parameters for optimisation, process control and scale up.

## 2. Methodology

**Figure 2:**
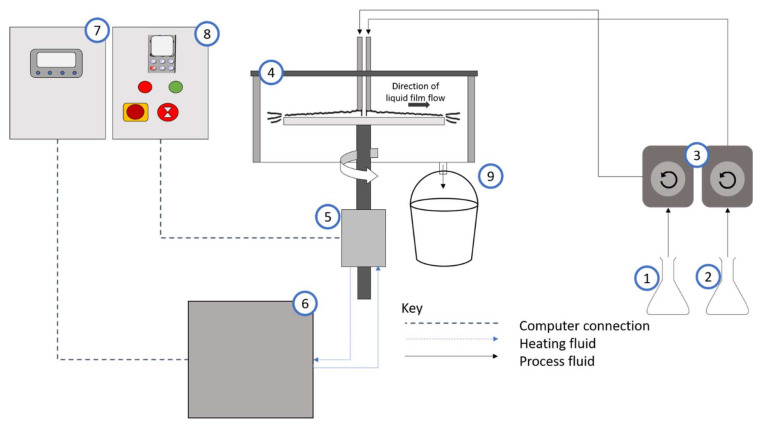
Schematic of spinning disc reactor (SDR) set-up: (**1**) Solute/ solvent; (**2**); antisolvent; (**3**) peristaltic pumps; (**4**) SDR; (**5**) motor; (**6**) heating tank; (**7**) temperature control unit; (**8**) SDR rotational control unit; (**9**) product outlet and receiver.

**Figure 3:**
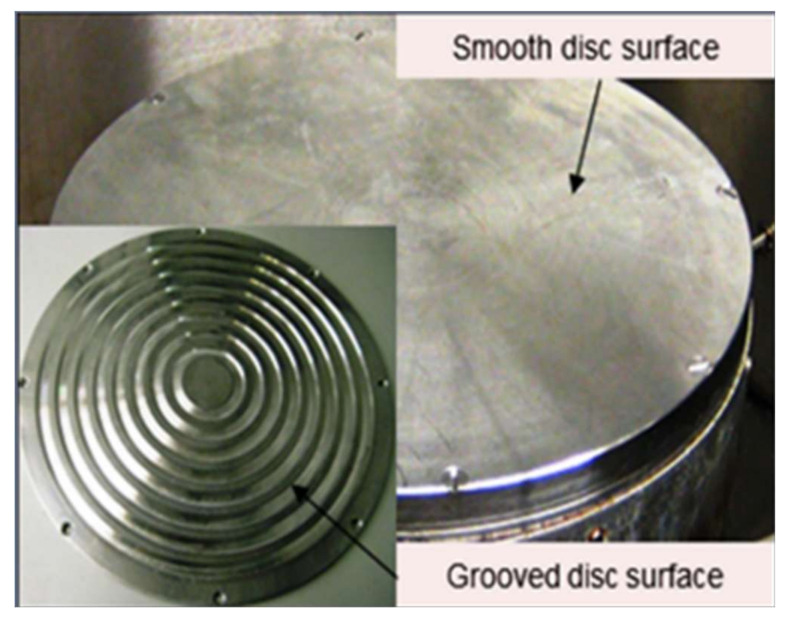
Grooved and smooth disc surfaces. Reproduced from [28], De Gruyter, 2019.

A schematic image of the spinning disc reactor set-up is presented in Figure 2. The spinning disc reactor consists of a horizontal 30 cm diameter stainless disc encased within a reactor housing. The samples are collected at an outlet connected to the reactor housing. Underneath the reactor is a water tank where water is heated to the required temperature (25 °C) and is circulated under the disc to control the temperature. The solute/solvent (starch/NaOH) mixture and the antisolvent (ethanol) are each fed to the reactor through a single-point feed distributor (internal diameter 1.5 mm) positioned in the centre, 2.3 cm above the surface of the disc. Experiments were conducted on a stainless-steel grooved disc with 8 concentric grooves and repeated on a stainless-steel smooth disc as shown in Figure 3.

The reagents, sodium hydroxide in pellet form and 99.8% absolute ethanol were purchased from Fisher Scientific, Loughborough, UK. Starch from corn was purchased from Sigma Aldrich, Gillingham, UK. 2% *w*/*v* starch in 0.5 M NaOH solution was prepared for use in these experiments. A full factorial design of experiments in the SDR, consisting of 3 factors and 3 levels was implemented based on the operating conditions given in Table 2. Each experimental run was carried out for a total of 60 s with samples collected at 20 s intervals. The samples were immediately quenched in deionised water to halt further precipitation of the nanoparticles.

Starch nanoparticles from the SDR were analysed using Dynamic Light Scattering (DLS-Mode Nano ZS Malvern instruments, Malvern, UK) technology to obtain intensity-based means. Further details of the methodology can be found in Sana et al. (2019) [28].

Regression analyses of the data have been carried out using Excel’s Data Analysis Add-on pack (Microsoft Excel, Microsoft Corporation, 2018) and QI Macros for Excel (QI Macros, KnowWare International, Inc., CO, USA, 2020).

### Dimensionless Numbers

Reynolds number for thin film flow in the SDR is typically defined as [60]:

(5) $$Re\;=\;\frac{2Q}{\pi \nu r}$$

It is commonly used to characterise liquid flow in the SDR as it is a function of flow rate, disc radius, and properties of the liquid. The Reynolds number criteria at which flow transitions from laminar to turbulent are defined as [60]:

Re<16: Smooth laminar flow
16≤Re<40: Small amplitude waves
40≤Re<80: Sinusoidal waves replaced by regular waves
80≤Re<1000–2000: Wavy-laminar flow
Re≥1000–2000: Turbulent flow regime

However, this particular definition of Reynolds number does not encompass the rotational aspect of the SDR. For this reason, another dimensionless number will also be incorporated into the model to characterise rotation of the disc. Two dimensionless groups will be considered: The rotational Reynolds number, *Re_ω_,* and the Rossby number, *Ro.* The merits and applicability of each are discussed below.

The rotational Reynolds number is a dimensionless number which may be used to describe the rotational aspect of flow on the film. Similar to the conventional Reynolds number, it is the ratio of inertial (centrifugal) to viscous forces and is expressed in the following form:

(6) $$Re_{\omega }\;=\;\frac{\omega r^{2}}{\nu }.$$

The rotational Reynolds number provides an alternative to the conventional Reynolds number for characterising flow regime in the reactor as *Re* does not take angular velocity into consideration [49,61]. Similarly, *Re_ω_* does not include flow rate in its expression. Ozar et al. (2003) states that both flow rate and disc speed play a major role in flow transition from laminar to turbulent [62]. Rotational Reynolds number criteria for categorising flow regimes in a spinning disc reactor are as follows [61]:

$Re_{\omega }$< 10^4^: Laminar regime
10^4^$\leq Re_{\omega }$<10^5^: Flow instabilities increase and flow is in transition to turbulent regime
$Re_{\omega }\geq$ 10^5^: Turbulent regime

Often the rotational Reynolds number is identified as the Taylor number, *Ta*. Saw et al. (1985) used the Taylor number along with the conventional Reynolds number to develop a predictive model for liquid film thickness on a rotating disc [63]. This was further applied by Khan (1986) and by Mohammadi (2014), the latter proposing that TiO_2_ particle size is directly proportional to the dimensionless form of liquid film thickness [64,65]. Their models assumed negligible Coriolis forces on the film, provided *Re*^2^/*Ta* is less than unity. For the present work, *Re*^2^/*Ta* is in the range of 10^−3^ to 10^−5^ at the edge of the disc, though tends to get greater towards the centre of the disc. As 90% of the disc has a value less than 1, it may be assumed that Coriolis forces are negligible in our experimental work.

Alternatively, the Rossby number has been used in previous work to characterise liquid flow on a spinning disc [66,67,68]. It is defined as the ratio of inertial (centrifugal) to Coriolis forces. The Rossby number is presented in Equation (7), where *u_i_* is the inlet velocity calculated from total flow rate of the antisolvent and solvent/solute streams. The Rossby number is estimated to lie in the range of 0.045 to 0.405 for the operating conditions used in the present work, indicating dominance of Coriolis forces over inertial when *Ro* < 1, whereas centrifugal forces dominate when *Ro* << 1 [67]. These values have been estimated at the edge of the disc in order to correlate with particle size measurements for particles collected at the edge of the disc. The Rossby number is often applied in combination with the Ekman number, *Ek*, as presented in Equation (8) [69]. It implies that for a small *Ro* value, *Ek* will be large in order to maintain an order of magnitude of 1. In circumstances where *Ro* << 1, the Ekman number is significantly greater to satisfy Equation (8), implying negligible Coriolis forces [70]. Based on this, the *Ro* values in the present work are considered to be within the region where Coriolis forces are negligible, as *Ek* >> 1 at the outer region of the disc [70]. As with the Rossby number, towards the centre of the disc, Ekman number too tends towards values where Coriolis forces come into play. *Ek* of less than 2 is where flow deviates from the centrifugal model [49]. For the current system, *Ek* is greater than 2 for the majority of the reactor.

(7) $$Ro\;=\;\frac{u_{i}}{\omega r}$$

(8) $$Ek\;=\;\frac{\nu }{\omega \delta^{2}}.$$

Finally, apart from the hydrodynamic parameters, we have also included the dimensionless supersaturation ratio term, *S*, in our empirical model to account for the effect of antisolvent to solvent flow ratio. *S* is defined in Equation (9), where C* is the solubility of starch which varies with the antisolvent to solvent ratio and the solute concentration, *C*, is kept constant at 2% *w*/*v* in all experiments.

(9) $$S\;=\;\frac{C}{C^{*}}$$

## 3. Results and Discussion

**Figure 4:**
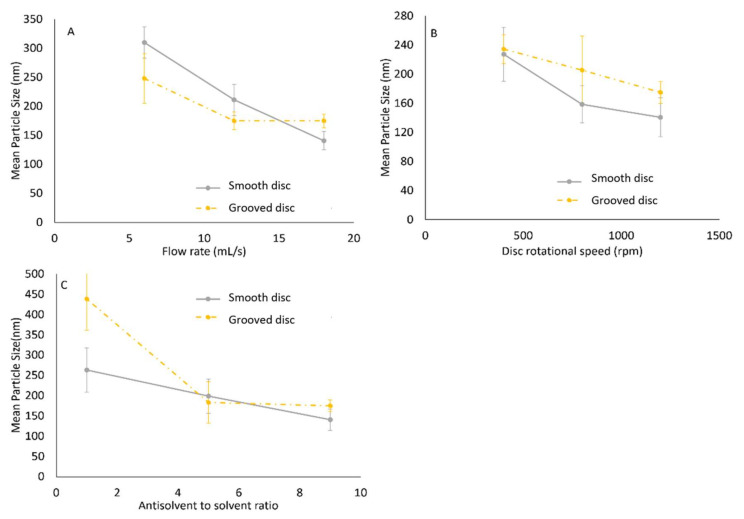
Effect of: (**A**) Flow rate (1200 rpm, 9:1 ratio); (**B**) disc rotational speed (18 mL/s, 9:1 ratio); (**C**) antisolvent to solvent ratio (1200 rpm and 18 mL/s) on particle size for smooth and grooved discs. Reproduced from [28], De Gruyter, 2019.

A selection of the mean particle sizes obtained from the DLS measurements is displayed in Figure 4 [28]. These correspond to varying conditions of flow rate, disc rotational speed and antisolvent to solvent ratio. In summary, the graphs show a reduction in particle size at increased conditions of flow rate, disc rotational speed and antisolvent to solvent ratio up until the central point. Further increase in the operational parameters leads to either an increase in particle size or no further change in particle size as a result of particle agglomeration or the poor mixing phenomenon at conditions of 1:1 antisolvent to solvent ratio and high flow rate/disc speed. Further discussion of the experimental results can be found in [28].

### 3.1. Model 1: Using Rotational Reynolds Number to Characterise Disc Rotational Speed

The particle size for starch nanoparticles can be represented in the form of Equation (10) for the rotational Reynolds number. This particular form of linear multiple regression has been selected for simplicity and has been used previously in precipitation systems to predict particle size [39,65].

(10) $$Particle\;size\;\left(\mathrm{microns}\right)\;=\;A\;Re^{a}Re_{\omega }{}^{b}S^{c}$$

where *A* (microns), *a*, *b* and *c* are coefficients of the regression model. The units for particle size are in microns rather than nanometres to avoid large values of coefficient *A* and to keep all coefficients roughly of similar magnitudes. Furthermore, with coefficient *A* and particle size being in microns ensures dimensional agreement is preserved.

The following models have been generated for the smooth and grooved discs:

Smooth disc:

(11) $$Particle\;size\;\left(\mathrm{microns}\right)\;=\;10^{0.32}Re^{-0.08}Re_{\omega }{}^{-0.13}S^{-0.03}$$

*R*^2^ = 0.933, *R*^2^ (adj.) = 0.913

Grooved disc:

(12) $$Particle\;size\;\left(\mathrm{microns}\right)\;=\;10^{0.24}Re^{-0.26}Re_{\omega }{}^{-0.08}S^{-0.01}$$

*R*^2^ = 0.930, *R*^2^ (adj.) = 0.909

The models are applicable for the following ranges, with *Re* and *Re_ω_* estimated at radial distances of 15 cm from the centre where the nanoparticles were collected and measured:

8.21≤Re≤52.4

$$3.04\times 10^{5}\leq Re_{\omega }\leq 1.94\times 10^{6}$$

116≤S≤1074

**Figure 5:**
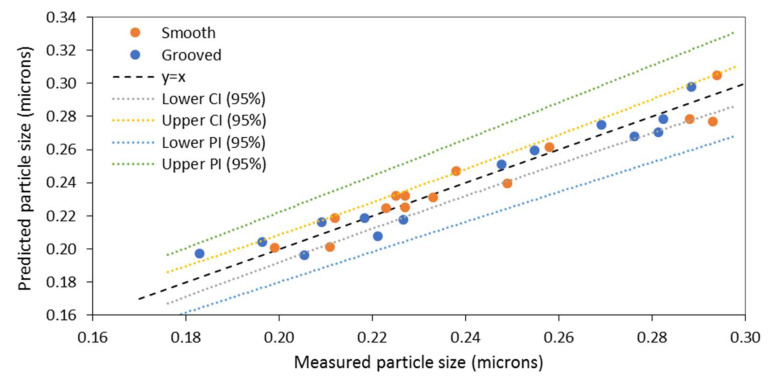
Comparison between predicted particle size and experimental particle size for smooth and grooved discs using the rotational Reynolds number (*Re_ω_*).

Figure 5 displays a comparison between the experimental data and data predicted from the models given in Equations (11) and (12). The negative sign of the coefficients in Equations (11) and (12) indicates a negative correlation between all the dimensionless parameters and particle size. Thus, it is predicted that an increase in *Re* would lead to a reduction in particle size. This would occur at high flow rates or low viscosities as described in Equation (5). Similarly, *Re_ω_* is greater at higher disc rotational speeds, leading to a reduction in particle size. An increase in initial supersaturation ratio also results in smaller particles, although the magnitudes of the coefficients in Equations (11) and (12) indicate that particle size is least influenced by initial supersaturation, *S*, than *Re* or *Re_ω_* for both disc textures studied.

According to the range of rotational Reynolds numbers encountered in the SDR, the flow is primarily in the transitional or turbulent regime, although the conventional Reynolds number, *Re*, suggests the flow regime falls between laminar and wavy-flow regimes. It is apparent from Equations (11) and (12) that the rotational Reynolds number, *Re_ω_*, hence disc rotational speed, is more significant for the smooth disc, whereas *Re,* hence flow rate is more influential for the grooved disc. One explanation for this difference would be that in the presence of grooves the flow regime is more likely to transition into turbulent flow at lower values of *Re_ω_* [61]. This would mean that the grooved disc is capable of achieving greater turbulence at lower disc rotational speeds, hence the grooved disc is influenced less by disc speed and more by flow rate. Supersaturation has the least impact on particle size, based on the small magnitude of the coefficient, especially for the grooved disc. This may be caused by the supersaturation values applied in the experimental work being more heavily weighted towards the higher end of the range; a larger range of values, particularly lower values of *S*, may very well show a greater dependency of particle size on supersaturation. Additionally, ideal mixing conditions provided by the grooves could also have contributed to minimising the influence of *S* on particle size through the uniform distribution of supersaturation at all values of supersaturation. It is also worth noting that results deemed to be outliers have been removed from the data set included in the model derivation, specifically, particle size attained at 1:1 ratio and 18 mL/s, where the occurrence of back-mixing was considered to be an issue in the reliability of the measured data [28]. *R*^2^ and adjusted *R*^2^ values are also presented in Equations (11) and (12) for the regression models. The values are greater than 0.9, indicating a good fit between the predictive model and the experimental results. The confidence intervals (CI) and prediction intervals (PI) displayed in the plots highlight the upper and lower limits of the regression model at a 95% confidence level. The confidence interval tells us that there is 95% certainty that the regression model lies within this interval, whereas the prediction interval tells us that we can be 95% confident that the next observation is likely to fall within this region. As most points lie within the bounds of the confidence and prediction intervals, it can be concluded that the regression model is a good fit to predict particle size.

### 3.2. Model 2: Using Rossby Number to Characterise Disc Rotational Speed

A regression model has been generated using the Rossby number to characterise disc rotation. As the Rossby number is a function of both inlet velocity and angular velocity (Equation (7)), the Rossby number will be applied in this model to characterise both flow rate and disc rotational speed. The following models have been obtained for the smooth and grooved discs at a 15 cm radial distance from the centre.

Smooth disc:

(13) $$Particle\;size\;\left(\mathrm{microns}\right)=\;10^{-0.37}Ro^{0.03}S^{-0.08}$$

*R*^2^ = 0.778, *R*^2^ (adj.) = 0.734

Grooved disc:

(14) $$Particle\;size\;\left(\mathrm{microns}\right)=\;10^{-0.36}Ro^{-0.06}S^{-0.12}$$

*R*^2^ = 0.854, *R*^2^ (adj.) = 0.830

The models are applicable for the following range:

0.045≤Ro≤0.405

116≤S≤1074

**Figure 6:**
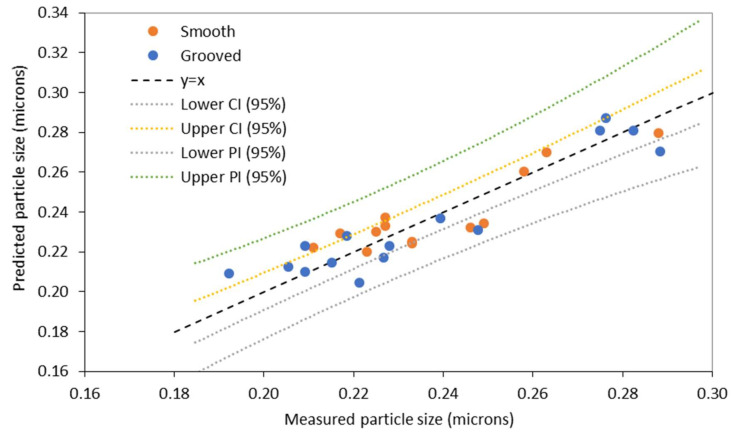
Comparison between predicted particle size and experimental particle size for smooth and grooved discs using Rossby number (*Ro*).

In Equation (14), the coefficient for the Rossby number has a negative sign for the model predicted for the grooved disc, implying that as the Rossby number increases, particle size decreases. An increase in *Ro* would be influenced by greater flow rate, which agrees with the previous model, suggesting that disc rotational speed has less of an effect on particle size on the grooved disc. On the smooth disc the opposite is true, indicated by the positive coefficient for the Rossby number, representing a greater dependence of particle size on disc rotational speed. A decrease in the Rossby number, caused by an increased disc rotational speed, would produce smaller sized particles on the smooth disc. However, because both flow rate and disc speed parameters are represented by the single dimensionless number, it is difficult to speculate on the exact relationship between the parameters and *Ro*. Furthermore, unlike the previous models, here supersaturation appears to have more of an impact on particle size, as implied by the larger coefficient. Figure 6 shows a comparison between the predicted and measured particle size values using the models given in Equations (13) and (14).

The confidence and prediction intervals at a 95% confidence level are also displayed in Figure 6. All points are within the PI and the majority within the CI. The values of *R*^2^ and *R*^2^ adjusted (Equations (13) and (14)) for the current regression model are also lower in comparison to the previous model. The Rossby model is composed of only two independent variables (*Ro* and *S*) unlike the previous model (Model 1) which has three independent variables, and often an increase in the number of independent variables in a multiple regression model leads to an increase *R*^2^ values, bringing them closer to 1. It also evident from the Rossby number expression (Equation (7)) that there are no parameters describing the physical properties of the fluid, such as viscosity, which is an important parameter when considering the flow of shear-thinning starch in the SDR. Equations (15) and (16) presents the regression model with the addition of Reynolds number to account for the viscosity of the fluid.

Smooth disc:

(15) $$Particle\;size\;\left(\mathrm{microns}\right)\;=\;10^{-0.24}Ro^{0.12}Re^{-0.23}S^{-0.01}$$

*R*^2^ = 0.905, *R*^2^ (adj.) = 0.87.

Grooved disc:

(16) $$Particle\;size\;\left(\mathrm{nm}\right)\;=\;10^{-0.02}Ro^{0.11}Re^{-0.34}S^{-0.01}$$

*R*^2^ = 0.913, *R*^2^ (adj.) = 0.890.

The regression models (Equations (15) and (16)) for both discs suggest a greater influence of flow rate on particle size as indicated by the larger magnitude of the Reynolds number coefficients. This contradicts the previous models obtained for the smooth disc. The coefficients for supersaturation are also smaller in comparison to the *Ro* model. However, this model has both *Ro* and *Re* measuring flow rate, introducing multicollinearity as both variables are highly correlated [71]. This is denoted by the sign change of the coefficient associated with *Ro* in Equation (16). Furthermore, the standard error associated with the regression coefficient for *Ro* increases from 0.02 to 0.05 upon the introduction of *Re*. However, this does not seem to be the case for the smooth disc, though often multicollinearity can go undetected [71]. Hence, the model using *Ro* alone is better to represent the current system.

### 3.3. Evaluation of the Models

Both the Rossby number and rotational Reynolds number models show good correlation between the predicted and measured particle size at a 95% confidence level, though the *Ro* model has lower values of *R*^2^ and *R*^2^ adjusted. The Rossby number only considers the liquid velocity at the entrance of the SDR and does not take account of the viscous forces on the disc, whereas both the rotational Reynolds number and Reynolds number for flow rate incorporate rheological effects through the viscosity term. Furthermore, above the stated range of Rossby numbers studied, Coriolis forces may begin to dominate and for the Nusselt model, and thus, Equations (1)–(3) to be valid, Coriolis forces must be negligible. This is true for the experimental conditions studied as $\nu \gg \omega \delta^{2}$, where δ is the film thickness [60].

Empirical model 1 provides a better fit of the data as indicated by the R^2^ values which are above 0.9 for both discs. To further distinguish between the two models, standard error for the models are presented in Table 3. The standard error represents the distance between data points and the regression line. Model 1 has slightly smaller standard error values, which indicates that the predicted values are closer to the measured values.

Additionally, both models have demonstrated that particle size is influenced more strongly by flow rate and disc rotational speed. Both these parameters impact shear rate on the disc through the following equation:

(17) γ˙ = ω2rν 3Qν2πω2r213.

Through greater shear, the degree of micromixing between the solvent/solute and the antisolvent increases and the collisions between particles escalate to form the critical nucleus, leading to a faster nucleation rate and therefore smaller nanoparticles.

The effect of antisolvent to solvent ratio, or supersaturation, has been considered low or negligible according to the first model. This is particularly the case on the grooved surface. However, the Rossby number model presents supersaturation as a significant parameter. Looking at the previously published experimental results [28], particle size is reduced at greater antisolvent to solvent ratios, though with the outliers removed (back-mixing at 18 mL/s, 1:1) the data used in model generation is narrowed, thus the impact of supersaturation at this point is less certain. Valente et al. (2012) also found that for the precipitation of polymer nanoparticles in a CJIM, antisolvent to solvent ratio had a lesser impact on particle size as a result of mixing efficiency decreasing at greater antisolvent to solvent ratios [39].

**Figure 7:**
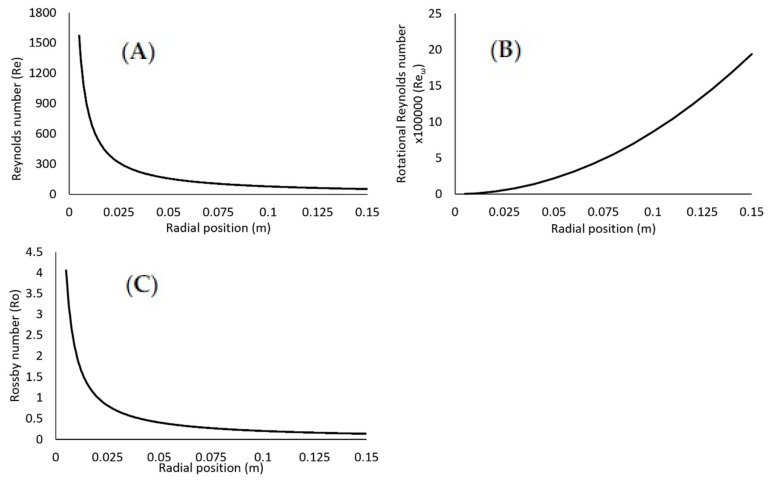
Effect of radial position on dimensionless parameters: (**A**) Reynolds number; (**B**) rotational Reynolds number; (**C**) Rossby number at 1200 rpm, 18 mL/s and 9:1 ratio.

**Figure 8:**
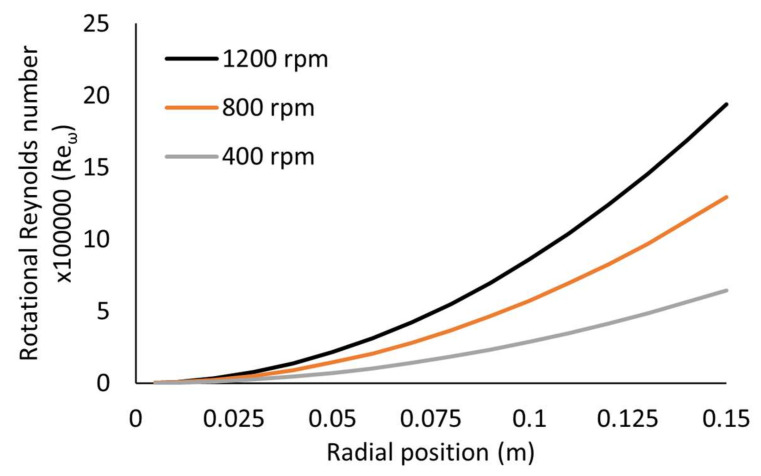
Effect of radial position on rotational Reynolds number at various disc speeds and 18 mL/s and 9:1 ratio.

Finally, the dimensionless quantities have been estimated for radial distances close to the edge of the disc, and as conditions such as film thickness vary along the radius of disc, the dimensionless numbers would be affected by the radius, *r*. The effect of radial position on the dimensionless numbers, *Re*, *Re_ω_* and *Ro* are presented in Figure 7 at conditions of 1200 rpm, 18 mL/s and 9:1 ratio. For example, rotational Reynolds number increases away from the centre of the disc, reaching a maximum value at the edge of the disc. The flow near the centre of the disc is initially in the laminar regime, with the transitional regime occurring between 0.03 and 0.01 m of the disc, and beyond that the flow is fully turbulent. By decreasing the disc speed (Figure 8), the turbulent regime occurs further away from the centre of the disc, and at 400 rpm the flow remains in the transitional regime towards the edge of the disc. This could have contributed to the production of larger particles at the lower disc speed. The Reynolds number also varies with radial position, evolving from a turbulent flow regime to a wavy regime towards the edge of the disc. Finally, Figure 7C shows a decline in the Rossby number with radial position, further indicating that Coriolis effects become negligible beyond roughly 0.025 m from the centre of the disc. However, the effect of radial distance on the dimensionless correlations have not been investigated further as nanoparticle samples have only been collected at the edge of the disc.

## 4. Conclusions

Empirical correlations have been developed relating the size of starch nanoparticles produced in the SDR to the key parameters: Flow rate, disc rotational speed and antisolvent to solvent ratio. Dimensionless numbers have been applied to characterise these parameters. Three regression models have been proposed through combinations of Reynolds number, rotational Reynolds number, Rossby number and dimensionless supersaturation. Correlations based on the rotational Reynolds number to characterise disc speed have indicated the closest agreement between measured and predicted particle sizes and have demonstrated that particle size is more influenced by flow rate and disc rotational speed than initial supersaturation.

## Figures and Tables

**Table 1:** Methods of starch nanoparticle production.

Technique	Starch Nanoparticle Production Process	Size	Ref.
Hydrolysis (top-down)	Acid hydrolysis	20–90 nm (SEM) 40–70 nm (TEM) 70–100 nm (TEM)	[19] [20] [21]
Physical methods (top-down)	Enzymatic hydrolysis	500 nm (DLS)	[22]
	Gamma radiation	20–30 nm (DLS)	[23]
	Ultrasonication	30–100 nm (SEM)	[24]
	High pressure homogenisation	<50 nm (TEM)	[25,26]
	Cold plasma and ultrasound	300–1000 nm (DLS)	[27]
Precipitation (bottom-up)	Solvent–antisolvent precipitation	50–100 nm (SEM) 150 nm (SEM) 130–400 nm (DLS) 140–450 nm (DLS)	[10] [18] [16] [28]

**Table 2:** Operating conditions for spinning disc reactor (SDR) experiments.

Factor	Low	Centre	High
Disc rotational speed (rpm)	400	800	1200
Total flow rate (mL/s)	6	12	18
Antisolvent to solvent ratio (vol/vol basis)	1:1	5:1	9:1

**Table 3:** Comparison of standard errors generated by the two empirical models.

Model	Standard Error
Model 1 $(Re_{\omega },Re,S$) Grooved disc	0.020
Model 1 $(Re_{\omega },Re,S$) Smooth disc	0.016
Model 2 $\left(Ro,S\right)$ Grooved disc	0.023
Model 2 $\left(Ro,S\right)$ Smooth disc	0.020

## Nomenclature:

C	Solute concentration (g solute/g solvent)
$C^{*}$	Equilibrium concentration (g solute/g solvent)
Q	Volumetric flow rate (m^3^ s^−1^)
r	Radial position from centre of disc (m)
$t_{\mathrm{res}}$	Mean disc residence time (s)
$u_{\mathrm{av}}$	Film thickness-averaged radial velocity on disc (m s^−1^)
$u_{i}$	Inlet velocity (m s^−1^)
$v_{r}$	Instantaneous disc radial velocity (m s^−1^)
Greek symbols	
δ	Liquid film thickness (m)
$\dot{\gamma }$	Shear rate (s^−1^)
ω	Angular velocity (rad s^−1^)
ν	Kinematic viscosity (m^2^ s^−1^)
Dimensionless numbers	
Ek	Ekman number, $Ek=\frac{\nu }{\omega \delta^{2}}$ (-)
Re	Reynolds number, $Re=\frac{2Q}{\pi \nu r}$ (-)
$Re_{\omega }$	Rotational Reynolds number, $Re_{\omega }=\frac{\omega r^{2}}{\nu }$ (-)
Ro	Rossby number, $Ro=\frac{u_{i}}{\omega r}$ (-)
S	Supersaturation $S=\frac{C}{C^{*}}$ (-)
Subscripts	
*i*	Inlet
*o*	Outlet
